# A review of research progress on periplasmic adaptor proteins in bacterial tripartite pumps

**DOI:** 10.3389/fmicb.2026.1859451

**Published:** 2026-06-19

**Authors:** Zhenyue Feng, Xianmin Huang, Yue Chen, Cailing Qiu, Defu Liu

**Affiliations:** 1 College of Agronomy and Life Sciences, Zhaotong University, Zhaotong, China; 2 Yunnan Engineering Research Center of Green Planting and Processing of Gastrodia, Zhaotong University, Zhaotong, China; 3 Yunnan Key Laboratory of Gastrodia and Fungi Symbiotic Biology, Zhaotong University, Zhaotong, China

**Keywords:** efflux pump inhibitors (EPIs), Gram-negative bacteria, multidrug efflux pumps, periplasmic adaptor proteins, tripartite pumps

## Abstract

Gram-negative bacteria exhibit higher intrinsic resistance to antimicrobial agents than Gram-positive bacteria, largely attributable to their efficient multidrug efflux pumps. Among these, tripartite pumps spanning the inner membrane, periplasm, and outer membrane are central to both intrinsic and acquired resistance. Periplasmic adaptor proteins (PAPs) serve as the core connectors of these systems. Once regarded merely as passive scaffolds, PAPs are now confirmed to function as dynamic allosteric couplers that mediate complex assembly and conformational coordination. Moving beyond structural descriptions, this review synthesizes recent advances regarding the structural architecture of PAPs across RND (Resistance-Nodulation-Division), MFS (Major Facilitator Superfamily), and ABC (ATP-Binding Cassette) superfamilies, highlighting their conserved domains and subtype-specific adaptations. We place special emphasis on the multifaceted roles of PAPs-from substrate recognition and environmental sensing to their surprising function as extracellular “public goods” in bacterial necrosignaling. Furthermore, we dissect the clinical epidemiology of PAP overexpression and evaluate the potential of targeting PAPs with novel inhibitors. By reframing PAPs as active regulatory hubs rather than static linkers, this review elucidates the assembly logic of tripartite efflux mechanisms and provides a theoretical basis for combating antimicrobial resistance.

## 1 Introduction

Antimicrobial resistance (AMR) causes millions of deaths worldwide (Theuretzbacher et al., 2025; Breijyeh et al., 2020; Datta and Gupta, 2019). Current forecasts suggest annual fatalities could reach 8.22 million by 2050 (GBD 2021 Antimicrobial Resistance Collaborators, 2024). The WHO’s 2024 priority list highlights drug-resistant Gram-negative bacteria—such as *Klebsiella pneumoniae*, *Acinetobacter baumannii*, and *Escherichia coli* - as critical threats (Sati et al., 2025). This status is largely due to their ability to use efflux pumps for antibiotic evasion (Macesic et al., 2025).

Beyond antibiotic resistance, these pumps also regulate virulence and biofilm formation (Nishino et al., 2006; Alav et al., 2018; Soto, 2013; Mbara et al., 2026). In Gram-negative bacteria, the double-membrane envelope acts as both a protective barrier and a transport bottleneck. To overcome this limitation, bacteria evolved tripartite pumps that span the inner membrane, periplasm, and outer membrane (Rouvier et al., 2025; Mohanty et al., 2021; Blair et al., 2014; Theuretzbacher et al., 2025; Manrique et al., 2023).

These pumps consist of three subunits: an inner membrane transporter (IMP), an outer membrane factor (OMF), and a PAP (Compagne et al., 2023; Chen et al., 2022; McNeil et al., 2019; Symmons et al., 2015). Research traditionally focuses on the IMPs and OMFs as the primary engines of transport (Li et al., 2015; Blanco et al., 2016; Lowrence et al., 2019). In this model, PAPs are often viewed as passive structural linkers (Du et al., 2018; Symmons et al., 2009). However, this view is changing. PAPs are now recognized as active allosteric couplers that transmit conformational energy from the IMP to the OMF (Zgurskaya et al., 2015).

Most reviews summarize the overall architecture of these pumps. Here, we focus specifically on PAPs. We discuss their domain structure and dynamic assembly, explaining how environmental factors like pH and cations fine-tune their function. We also cover the subunit interchangeability of PAPs and their role in periplasmic necrosignal, including the “public goods” hypothesis. Finally, we assess the potential of PAPs as targets for next-generation efflux pump inhibitors (EPI) (Hinchliffe et al., 2013; Fernández and Hancock, 2012; Alav et al., 2018; Wang et al., 2016a). By reframing PAPs as dynamic regulators rather than static scaffolds, we aim to deepen our understanding of resistance mechanisms and guide future therapeutic strategies.

## 2 Bacterial tripartite pumps

Periplasmic adaptor proteins do not act alone; they function as critical adaptors in multicomponent complexes. In Gram-negative bacteria, many clinically relevant efflux systems form tripartite pumps that span the inner membrane, periplasm, and outer membrane. By bridging IMPs and OMFs, PAPs coordinate substrate export across the cell envelope—and it is in this context that their regulatory roles become fully apparent.

**FIGURE 1:**
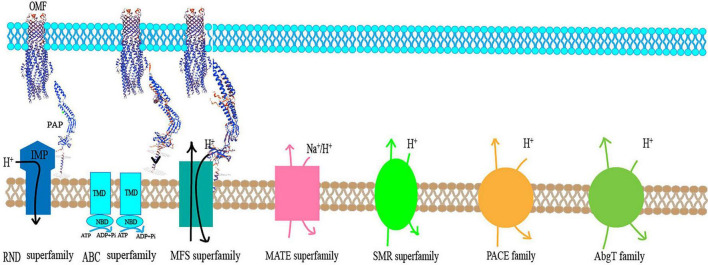
Seven families of multidrug transport proteins. The diagram illustrates the key families of efflux transporters responsible for multidrug resistance in Gram-negative bacteria, spanning the inner (brown) and outer (cyan) membranes. From left to right: RND superfamily, ABC superfamily, MFS superfamily, MATE superfamily, SMR superfamily, PACE family, AbgT family.

Bacterial efflux pumps are mainly classified into primary transporters that utilize ATP hydrolysis for energy (ATP-Binding Cassette, ABC) and secondary transporters that rely on the proton motive force for energy supply (Kumar and Varela, 2012; Chitsaz and Brown, 2017). Secondary transporters can be further classified into six superfamilies, including RND (Resistance-Nodulation-Division), MFS (Major Facilitator Superfamily), and MATE (Multidrug And Toxic Compound Extrusion), among which certain members possess the ability to assemble with PAPs and OMFs to form tripartite pumps (Figure 1; Zhao et al., 2022). Importantly, beyond secondary transporters, some ABC transporters (such as MacB) can also form functional tripartite pumps via PAPs and OMFs (Zgurskaya et al., 2015). In these complexes, PAPs serve as essential couplers that link the IMP to the OMF and help coordinate assembly and function.

**FIGURE 2:**
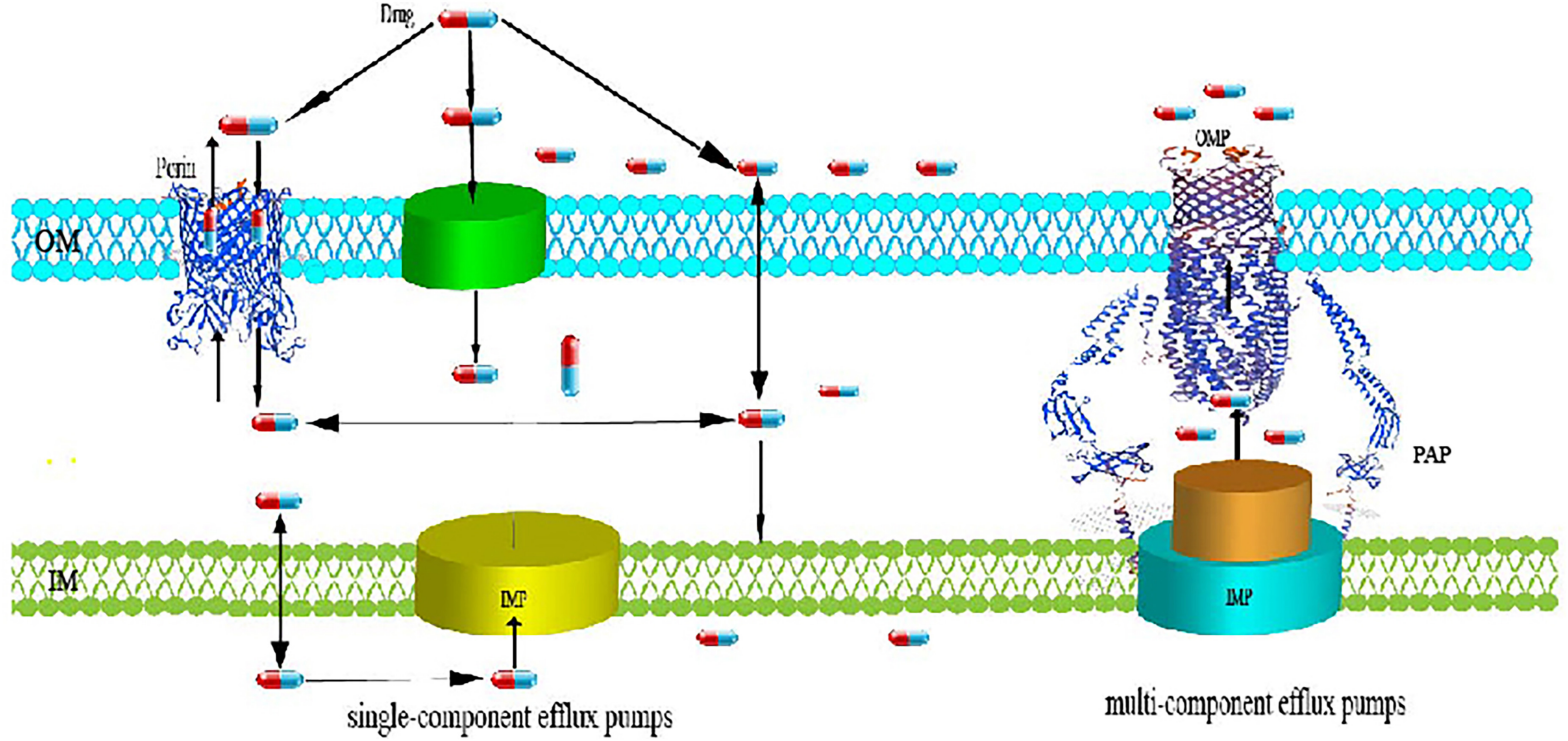
Single-component pumps and multi-component (tripartite) pumps. Efflux pumps in Gram-negative bacteria are categorized into single-component and multi-component systems. This figure based on Dinh et al. (1994). Single-component pumps transport substrates into the periplasm, while multi-component (tripartite) pumps span both inner and outer membranes. These tripartite systems form a continuous channel across the bacterial envelope, powered by proton gradients or ATP, to extrude substrates from the cytoplasm to the extracellular environment (Zgurskaya et al., 2018).

At the operational level, these tripartite pumps operate as multi-component pumps spanning the entire cell envelope. In Gram-negative bacteria, this is in stark contrast to single-component transporters that export substrates primarily into the periplasm (Figure 2; Dinh et al., 1994). The structure of the tripartite architecture consists of three basic components -IMP, PAP, and OMF- which together form a continuous substrate transport pathway to the extracellular environment (Zgurskaya et al., 2018). Functionally, these tripartite pumps operate as multi-component systems that span the entire cell envelope. OMF channels such as TolC often undergo dynamic switching between “open” and “closed” conformations: in the resting state, the channel is in a “closed” state to prevent substrate movement; the transition to the “open” state is triggered by PAPs. These adaptor proteins are indispensable for stabilizing transmembrane assembly and promoting substrate export via OMFs (Zgurskaya et al., 2015).

Based on the assembly mechanism and energy utilization, bacterial tripartite pumps include three major families: RND-, MFS- and ABC-type. RND and MFS complexes are typically PMF-driven secondary transport systems, whereas ABC-type tripartite pumps such as MacAB-TolC use ATP hydrolysis as the energy source (Neuberger et al., 2018).

The RND pumps, represented by the AcrAB-TolC complex, are the most common and functionally crucial efflux systems in the *Enterobacteriaceae*. This tripartite pump consists of the IMP AcrB, the PAP AcrA, and the widely used OMF TolC, forming a PMF-powered tripartite pump. AcrAB-TolC exports a broad range of substrates, including multiple types of antibiotics, bile salts, and other xenobiotics, contributing substantially to baseline drug tolerance and to the evolution of higher-level resistance (Kumar and Varela, 2012).

In contrast to the RND-type pumps, MFS pumps constitute another important proton-driven secondary transport system in Gram-negative bacteria. MFS transporters assemble with cognate PAPs and OMFs to form a tripartite pump. The EmrAB-TolC is the most thoroughly studied MFS pump and is the classic representative of this family (Yousefian et al., 2021). This complex comprises the IMP EmrB, the PAP EmrA, and the shared OMF TolC, forming a complete trans-envelope transport pathway. Unlike the RND pumps that can recognize a wide variety of structurally distinct substrates and exhibit high substrate promiscuity, the substrate specificity range of EmrAB-TolC is narrower and more specific (Elkins and Mullis, 2007).

ABC-type tripartite pumps such as MacAB-TolC use ATP hydrolysis to drive export. MacAB-TolC is best known for contributing to macrolide resistance, and it has also been linked to the export or secretion of non-antibiotic substrates, including certain virulence-associated factors in specific pathogens (Zgurskaya et al., 2015). Besides regulating antibiotic resistance, this specialized tripartite pump is also involved in the secretion of bacterial virulence factors and the regulation of the pathogenicity of Gram-negative bacteria.

The coexistence of RND-, MFS-, and ABC-type tripartite pumps within a single cell broadens the functional repertoire of efflux, enhancing bacterial adaptation to diverse chemical stresses and shaping drug tolerance profiles.

## 3 Structure of PAPs

### 3.1 Common structural modules

In Gram-negative bacteria, the PAP protein serves as the core auxiliary component of the tripartite pump system. Its overall spatial conformation, composition of conserved domains, and molecular mechanism are highly conserved among different bacterial species. This conservation serves as the structural basis for maintaining the common assembly mode and efflux function of various Gram-negative bacterial tripartite pumps, and is also the core prerequisite for PAP protein to mediate the assembly of efflux pump complexes across bacterial species and exert transport functions (Symmons et al., 2015).

Periplasmic adaptor proteins are multi-domain proteins. Analysis of 236 PAP structures by the Zgurskaya group revealed that although the amino acid sequences of PAPs are relatively less conserved and their protein lengths vary significantly, their three-dimensional structures are highly conserved. They typically present an extended linear conformation that extends from the inner membrane to the periplasmic space, where they engage OMFs (Zgurskaya et al., 2009). Importantly, the first 60 amino acids at the N-terminus and the last 100 amino acids at the C-terminus form conserved hydrophobic regions, which can interact with components of both the inner and outer membranes (McNeil et al., 2019).

**FIGURE 3:**
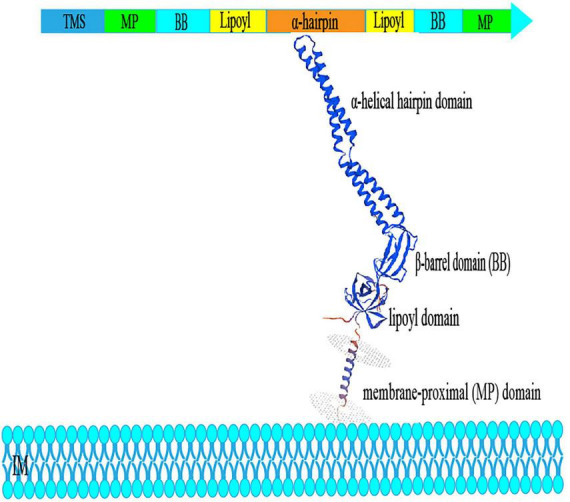
Structure diagram of periplasmic adaptor proteins. The domains include the membrane-proximal domain (MPD), the β-barrel domain (BB), the lipoyl domain, and the α-helical hairpin domain. The N-terminus encodes a small portion of the MPD and the BB domain, half of the lipoyl domain, and one α-helix of the α-helical hairpin domain.

Most PAPs contain four distinct domains between their N- and C-termini, with their periplasmic regions forming elongated, sickle-like conformations. These domains comprise a membrane-proximal domain (MPD), a β-barrel domain, a lipoyl domain, and an α-helical hairpin domain (Figure 3; Alav et al., 2022; Symmons et al., 2015). The MPD mediates binding to IMPs through specific motifs, which is essential for proper complex assembly. In addition, the MPD contributes to substrate passage and helps stabilize the active efflux complex. The β-barrel region, characterized by a flexible hinge made up of six antiparallel β-strands, plays a crucial role in facilitating conformational changes, relaying signals, and ensuring the stability of complexes (Mikolosko et al., 2006; McNeil et al., 2019). The lipoyl region acts as a structural framework, transduces conformational signals from the MPD via either homo- or heterotrimerization, and drives the ordered rearrangement of the downstream α-helical hairpin region (Alav et al., 2022; Lewis et al., 2023). This α-helical hairpin region, which consists of antiparallel α-helices, engages specifically with outer membrane components (such as TolC) and functions as the key interface for tripartite pump assembly (Alav et al., 2022). Flexible linkers connect these domains, providing the conformational flexibility required for energy transfer and functional coordination (Symmons et al., 2015).

In *E. coli*, the periplasmic pH fluctuates with external conditions, and the interplay between Mg^2 +^ availability and pH governs the structural dynamics of AcrA. Under acidic conditions, Mg^2 +^ depletion promotes structural flexibility, whereas Mg^2 +^ binding stabilizes an ordered conformation. Central to this regulation is His285; its protonation state remodels critical hydrogen-bond networks, sustaining pump activity across the physiological pH range of 5–8. Furthermore, the β-barrel domain acts as the functional core of AcrA, mediating the interdomain crosstalk required for signal transduction (Lewis et al., 2025). Beyond Mg^2 +^, cations such as Zn^2 +^, Cu^+^, and Ag^+^ also modulate efflux by engaging PAPs and inducing conformational rearrangements (De Angelis et al., 2010; Bagai et al., 2007).

The CusCFBA system exemplifies a distinct regulatory paradigm. Its PAP, CusB, binds Ag(I) with high affinity, and this interaction alone drives the conformational transition required for pump activation (Su et al., 2009). Thus, in this context, cations function dually as transport substrates and allosteric effectors.

These cases highlight the diverse structural plasticity among PAPs. While AcrA responds broadly to physicochemical cues, CusB is tuned by substrate-specific allosty. Other subfamilies, such as MacA and MdtA, appear to lack such direct structural tuning; instead, they adapt to stress primarily through transcriptional reprogramming (see Section“5.6 Functional modulation by environmental signals”). Consequently, rather than relying on a single conserved sensory mechanism, distinct PAP families have evolved specialized strategies to perceive and integrate environmental signals.

### 3.2 The structural commonalities and subtype differences of PAPs in the RND family

In the RND efflux superfamily, the representative PAPs, *E.coli* AcrA and *Pseudomonas aeruginosa* MexA, share a highly conserved elongated sickle-shaped four-domain architecture consisting of the MPD, β-barrel domain, lipoyl domain, and α-helical hairpin domain (Higgins et al., 2004; Symmons et al., 2009). Both proteins are N-terminally modified as lipoproteins and anchored to the inner membrane. They utilize their MPD and β-barrel domains as structural scaffolds to interact with cognate RND transporters (AcrB and MexB), while employing the α-helical hairpin domain to bind and induce the opening of outer membrane channels (TolC and OprM) (Zgurskaya and Nikaido, 1999; Higgins et al., 2004). The two proteins exhibit minor structural differences. The α-hairpin of AcrA is seven residues longer than that of MexA, and the connecting loop of AcrA shows greater conformational flexibility, whereas MexA adopts a more rigid crystallographic conformation (Higgins et al., 2004). Despite their high structural homology, AcrA and MexA fail to functionally complement one another in vivo. For instance, heterologous expression of mexA in a ΔacrA *E. coli* strain cannot restore bacterial drug resistance. This functional specificity is determined by subtle structural discrepancies at the interaction interfaces between PAPs and their cognate RND transporters and outer membrane channels (Symmons et al., 2009).

### 3.3 Structural commonalities and subtype differences of PAPs in the ABC superfamily

Within the ABC superfamily, PAPs such as MacA (drug efflux) and HlyD (secretion) share a conserved architecture built around MPD, β-barrel, lipoyl, and α-helical hairpin domains. Both proteins are anchored to the inner membrane, MacA via an N-terminal uncleaved signal peptide and HlyD via a transmembrane helix, and can associate with OMFs to form extended periplasmic complexes. Despite these shared features, key topological and functional distinctions exist between MacA and HlyD. MacA relies on an N-terminal uncleaved signal peptide for membrane anchoring and lacks a cytoplasmic domain (Fitzpatrick et al., 2017; Greene et al., 2018; Zhao et al., 2022). In contrast, HlyD contains an N-terminal transmembrane helix and a large periplasmic domain. This domain consists of two subdomains (D1 and D2), adopts a characteristic “head-stalk” architecture, which distinguishes its overall conformation markedly from that of AcrA. Unlike MacA, which functions mainly as a passive scaffold, HlyD possesses a small N-terminal cytoplasmic domain that binds the C-terminal signal peptide of substrates such as HlyA, enabling direct substrate sensing (Alav et al., 2021a).

ABC-type PAPs and RND-family PAPs (such as AcrA and MexA) show fundamental divergence in membrane anchoring strategies, assembly mechanisms, and functional structural elements. Firstly, the RND-type PAPs are anchored to the periplasmic side of the inner membrane via N-terminal lipid modification following signal peptide cleavage, whereas the ABC-type PAPs are directly integrated into the membrane through transmembrane helices. Secondly, the assembly of MacAB-TolC begins with the formation of the MacA-TolC subcomplex, followed by recruitment of the inner membrane ATPase MacB. In contrast, RND-type tripartite pumps (such as AcrAB-TolC) assemble in a defined order: the AcrA–AcrB subcomplex forms first, and TolC is subsequently recruited. Moreover, a unique “gate ring” formed by the conserved residue Gln209 in the MacA lipoyl domain can act as a “one-way valve” to prevent substrate backflow. This structural feature has not been observed in RND-type PAPs, reflecting the different evolutionary strategies of ABC and RND pumps to prevent channel leakiness (Higgins et al., 2004; Symmons et al., 2009; Huo et al., 2025).

### 3.4 The structural commonalities and subtype differences of PAPs in the MFS

Among the three-component efflux pumps of the MFS superfamily, the representative PAP EmrA exhibits distinct structural features that are significantly different from those of the RND and ABC family PAPs. Its core architecture consists of three linearly arranged domains, the β-barrel domain, the lipoyl domain, and a very long α-helical hairpin domain, but it clearly lacks the MPD that is commonly present in the other two types of PAPs. EmrA directly inserts into the inner membrane via an N-terminal single transmembrane helix and the β-barrel domain contains a conserved long disordered loop. This loop is considered a structural adaptation in MFS pumps that compensates for the short periplasmic extension of their IMPs (such as EmrB) (Hinchliffe et al., 2014).

Comparative analysis reveals fundamental differences between EmrA and the PAPs of both the RND and ABC families. The primary distinction lies in the presence or absence of the MPD and the resulting geometric shape adaptation: Both the RND and ABC PAPs have the MPD and thereby interact with the periplasmic region of their respective IMPs. In contrast, EmrA lacks the MPD entirely. This absence correlates with the structural feature of its cognate transporter (EmrB), which possesses a limited periplasmic domain. Consequently, its α-helical hairpin is significantly longer than the adaptor proteins of the RND and ABC families to compensate for the transmembrane distance. In terms of membrane anchoring mechanism, both EmrA and the PAPs of the ABC family insert through transmembrane helices into the inner membrane, while the PAPs of the RND family are anchored by N-terminal lipid modification (Hinchliffe et al., 2014; Greene et al., 2018; Higgins et al., 2004).

## 4 Interactions and assembly of the tripartite pump

Structural characterization of PAPs laid the foundation for understanding their functional mechanisms. Although the conserved domain architecture of PAP provides a static blueprint, the dynamic assembly and signal transduction process of the tripartite pump hinges on interactions among these structural modules.

The precise function of PAPs in the transport cycle remains under investigation. Alav et al. elucidate a streamlined mechanism based on recent insights into the MexAB-OprM and TriABC-OpmH systems in *P. aeruginosa*, along with necessary adjustments to the initial allosteric model of the *E. coli* AcrAB-TolC system (Lau et al., 2007). This model emphasizes changes in the affinity of interprotomer binding sites for PAP2 during the conformational cycling of RND transporters. It reveals a two-way communication system between RND transporters and PAPs, where the distinct binding sites on RND pumps lead to the formation of two different PAP conformers, known as PAP1 and PAP2. The initial phase involves the upward transmission of signals from RND to OMFs via PAP1. PAP1 not only aids in delivering drugs to the transporter’s proximal binding site but also detects the drugs’ movement toward the deeper binding site, a transition associated with the shift from the L state to the T state. The subsequent phase features downward signal transmission, triggered by the interaction of PAP2 with the OMF. In this context, PAP2 is crucial as it ensures effective docking with OMFs, initiates the conformational reset of RND transporters through the changes it transmits, and facilitates drug release by stabilizing the transition from the T state to the C state and aiding the conversion from the C state to the O state, thereby completing the efflux cycle (Alav et al., 2021b).

In addition to bidirectional signaling, the relationship between PAPs and OMFs demonstrates notable adaptability. In Gram-negative bacteria, PAPs and RND transporters that are part of the same operon usually work together, yet the regulatory mechanisms that dictate the interactions between PAPs and OMFs are remarkably flexible. Interestingly, different PAPs can associate with a single OMF, such as TolC (Boyer et al., 2022). PAPs dock with OMFs via their α-helical hairpin domains (Koronakis et al., 2004). This interaction creates a pathway for substrates to cross the outer membrane (Zgurskaya et al., 2009).

### 4.1 PAP–IMP–OMF interactions

The exact stoichiometry and assembly process of the RND-PAP-OMF tripartite pump are still not fully understood. At the subunit level, research conducted both *in vivo* and *in vitro* indicates that the functional unit of PAPs is a dimer. Strong evidence supporting the dimeric nature of PAPs as a functional unit is derived from investigations into the triclosan-specific transport complex TriABC-OpmH in *P. aeruginosa*. This RND-type transporter is known to consist of two PAPs, namely TriA and TriB (Fabre et al., 2021). These two distinct PAPs are crucial for the functional diversity of the TriC transporter. Although TriA and TriB share only 36% sequence identity, they both possess the essential characteristics of PAPs necessary for recognition (Alav et al., 2021b). Additionally, within the TriABC-OpmH complex, the two PAP subunits (TriA and TriB) have specific roles: TriA binds to the IMP while TriB interacts with OMF, with TriA primarily stabilizing the interaction with OMF and TriB being vital for activating the TriC pump (Fabre et al., 2021). Nonetheless, the mechanism by which the hexameric ring-like structure assigns these functions after assembly is still not clear.

### 4.2 Stoichiometry of the tripartite pumps

Initial models of tripartite assembly proposed a stoichiometric ratio of 3:3:3; however, this configuration complicates the understanding of how the distinct PAP members TriA and TriB interact independently with TriC and OpmH to create an efflux channel. Since both PAPs are crucial for the pump’s operation (Weeks et al., 2015), emerging evidence has led to a shift in consensus toward a 3:(3+3):3 assembly configuration (Alav et al., 2021b; Du et al., 2014). This shift is bolstered by observations such as RND transporters linked to two different PAPs (for instance, TriABC-OpmH) and the confirmed functionality of tandem-fusion AcrA constructs (Athar et al., 2023). Furthermore, *in vivo* cross-linking experiments focusing on both interprotomer and intraprotomer grooves of the OMF MtrE support the 3:6:3 stoichiometry of the OMF-PAP-RND complex (Mikolosko et al., 2006).

Although there have been substantial recent insights regarding the structure and function of all elements within the tripartite pump system, the arrangement, ratios, and interaction specifics of its subunits remain ambiguous and controversial (Du et al., 2014).

### 4.3 Dynamic assembly of tripartite pumps

External stress factors act as signals that initiate the assembly of tripartite pumps. In this context, PAPs function as key “sensors.” For example, in *E. coli*, the PAP CusB directly detects metal ions, triggering structural rearrangements that facilitate the rapid assembly of the CusCBA complex. This process occurs even before the metal-resistance *cus* operon is transcriptionally activated, establishing a pre-emptive efflux-ready state. This dynamic assembly, mediated by PAPs, maintains a balance between effective substrate removal and the adaptability of the periplasm, ensuring that the pump complex assembles only when needed, thereby avoiding unnecessary energy expenditure (Santiago et al., 2017; Chen et al., 2022; Shi et al., 2019).

The interactions among the component proteins and the adaptability of the *E. coli* AcrAB-TolC efflux pump have been investigated by Chen et al. (2022) who employed electron cryo-tomography to determine the structure of this pump in its natural environment. Their findings demonstrated that the N-termini and C-termini of the six AcrA protomers can exist in two different states: one in which they interact only with AcrB, and another in which they interact with both AcrB and the inner membrane. This suggests that AcrA protomers can anchor in the inner membrane, bind to AcrB, interact with TolC, and make contact with the peptidoglycan layer. These interactions enable conformational signals to be transmitted from inner-membrane AcrB to outer-membrane TolC. The researchers proposed a model that explains the active efflux mechanism of the AcrAB-TolC pump: drug binding to AcrB triggers a conformational shift in the AcrB porter domain, which is then transmitted to TolC through the AcrA-TolC interface. This conformational movement in the AcrB domain drives further conformational rearrangements in the AcrA assembly, leading to twisting of AcrA protomers and the subsequent opening of the TolC channel. Furthermore, compared with *in vitro* structures, the MPD of AcrA *in situ* undergoes an 8-degree rotation and interacts with the membrane, underscoring the importance of lipid-protein interactions in maintaining the pump’s structural integrity and flexibility (Chen et al., 2022).

Initially, it was believed that AcrA played an essential role in establishing a sealed connection between AcrB and TolC. Early *in vitro* work by Tikhonova et al. (2011) reported the formation of binary AcrB-TolC complexes. However, latest high-resolution structural evidence revealed that the periplasmic domain of AcrB, albeit with a lengthy sequence, cannot physically span the remaining gap toward the TolC channel entrance. For this reason, the assembly of a fully sealed and optimally functional efflux channel relies on AcrA (Chen et al., 2022; Singh et al., 2024). Interestingly, structural plasticity allows for the formation of binary complexes in the absence of one component. For instance, the AcrA-TolC binary complex can expel periplasmic substrates such as malachite green, albeit with significantly lower efficiency than the intact tripartite system. This suggests that while the tripartite complex ensures a tightly sealed channel, the binary interaction serves as a secondary efflux route (Bodrenko et al., 2023; Singh et al., 2024). These dimeric complexes can directly expel periplasmic substances from the cell. For instance, significant efflux of malachite green was observed in the Δ*acrA* single mutant (expressing AcrA and TolC), although at lower efficiency than the intact AcrAB-TolC tripartite complex. This difference may arise because AcrA contributes to forming a tightly sealed channel within the tripartite complex-a feature absent in dimeric assemblies-potentially leading to leakage of malachite green back into the periplasm (Singh et al., 2024).

Following the defined assembly sequence described above, the MacA-TolC subcomplex does not exist as a static structure, but undergoes dynamic reversible rearrangement in vivo. Unlike the passive compensatory response observed to component loss in the AcrAB-TolC system, PAPs in other tripartite pump systems exhibit a more proactive mode of action. Notably, research by Huo et al. (2025) on the *E. coli* MacAB-TolC system revealed a dynamic assembly mechanism centered on the MacA-TolC subcomplex. While structural studies confirm that MacA forms a stable hexameric funnel with TolC (Yum et al., 2009; Fitzpatrick et al., 2017), Huo et al. demonstrated that this pre-assembled “reservoir” is not static. Instead, MacA subunits can undergo reversible dissociation from the complex and are subsequently recycled to facilitate the recruitment of new MacB transporters. This “shuttling” mechanism allows the limited pool of MacA to efficiently engage with the abundant MacB and TolC, thereby optimizing substrate efflux while minimizing unnecessary ATP consumption (Huo et al., 2025).

### 4.4 Species-specific assembly and regulatory logic of tripartite pumps

Although the PAP has a highly conserved “bridging” function in structure, different Gram-negative pathogenic bacteria exhibit distinct protein assembly preferences when constructing tripartite pumps. *E. coli* and *Salmonella enterica* mainly rely on the classic AcrAB-TolC system (Duval and Lister, 2013), whereas *P. aeruginosa* has evolved an extensive Mex family system. In addition to the core MexAB-OprM, it can also flexibly pair with various outer membrane proteins such as OprM, OprA, or OpmB through MexXY (Wu et al., 2024). *A. Baumannii* possesses unique AdeABC and AdeIJK systems (Lee et al., 2024), forming an independent assembly lineage; while *K. pneumoniae* simultaneously utilizes the chromosome-encoded AcrAB-TolC and plasmid-borne OqxAB-TolC system (Wang et al., 2016b), a mixed assembly strategy that significantly accelerates the spread of clinical resistance.

At the genetic regulation level, different bacterial species employ distinct strategies to control the functional expression of PAP. *E. coli* and *S. enterica* utilize MarA/SoxS/Rob and other AraC family global regulatory factors to integrate oxidative stress and chemical stress signals, uniformly up-regulating AcrAB-TolC (Duval and Lister, 2013). The regulation in *P. aeruginosa* is more complex. In addition to the local repression of MexR on MexAB-OprM (Wu et al., 2024), MexXY is finely tuned through multiple independent and intersecting pathways such as MexZ-ArmZ (ribosomal stress), ParRS (cationic peptide sensing), and AmgRS (envelope stress) (Wu et al., 2024). In *A. baumannii*, AdeABC is mainly regulated by the AdeRS two-component system (TCS). In clinical strains, mutations in the dimerization domain of the AdeS sensor kinase (e.g., G186V, S188F) impair its normal function, leading to dysregulated and aberrantly high expression of the efflux pump (Lee et al., 2024). In *K. pneumoniae*, RamA has been confirmed to directly up-regulate AcrAB and OqxAB simultaneously, revealing a unique “crosstalk” mechanism between the efflux pump systems of this bacterium (Wang et al., 2016b).

This species-specific assembly and regulation logic directly determines the clinical drug resistance phenotypes and evolutionary directions of various pathogenic bacteria. *P. aeruginosa*, through its complex Mex family regulatory network, especially the inherent resistance mechanism of MexXY to aminoglycosides (Poole, 2005), becomes a major cause of refractory infections; while *K. pneumoniae* achieves rapid horizontal transfer of drug resistance genes within the Enterobacteriaceae family through the plasmid-carrying OqxAB systems and its global regulation by RamA (Vega et al., 2011). *A. baumannii* acquires a stable resistance phenotype against carbapenem antibiotics through frequent mutations in the AdeRS two-component system (Aranda et al., 2010). In summary, understanding these assembly fingerprints and regulatory circuits based on species-specificity is a key prerequisite for developing EPIs targeting specific pathogens and overcoming multidrug resistance.

## 5 Functional versatility of PAPs

Although the preceding section has detailed the assembly complexity and stringent stoichiometry required for tripartite pump formation, these roles only constitute the fundamental function of PAPs. Beyond acting as static structural scaffolds, PAPs possess extensive functional versatility and act as dynamic intermediates that transduce environmental signals into conformational alterations. This section addresses the multifunctional characteristics of PAPs, with a primary focus on their essential roles as structural linkers and signal transducers within efflux complexes.

### 5.1 Structural scaffolding and adaptive assembly

Periplasmic adaptor proteins play a vital role in the assembly of tripartite pump systems in Gram-negative bacteria, acting as essential structural connectors between IMPs - including those from the RND, ABC, and MFS families - and OMFs such as TolC and OprM (Bodrenko et al., 2023; Hinchliffe et al., 2013). Instead of simply serving as passive connectors, PAPs function as dynamic coordinators: they establish a versatile architectural framework that facilitates the assembly and conformational transitions of the entire system, thereby enabling the formation of functional efflux channels that span the cell envelope (Alav et al., 2021b). By binding to specific domains, PAPs ensure the correct assembly, stability, and energetic coupling within the pump complex. They mediate the linkage of the tripartite components through distinct structural motifs that govern interaction fidelity and functional coordination (Symmons et al., 2015).

The assembly of tripartite pumps represents a key adaptive response to environmental fluctuations. Central to this process, PAPs facilitate the rapid formation of trans-envelope efflux channels, enabling the efficient efflux of a broad spectrum of substrates, including antibiotics, disinfectants, dyes, and host-derived molecules such as bile salts and antimicrobial peptides. This inducible assembly is critical for bacterial resilience against antimicrobial stress (Santiago et al., 2017; Alav et al., 2021b).

### 5.2 Allosteric signal transduction

Periplasmic adaptor proteins are not merely structural components for tripartite efflux complex assembly; they also act as key signal transducers that couple transmembrane signaling to pump gating. PAPs interact tightly with specific subdomains of IMPs through their MPD and β-barrel domain. These interactions support complex assembly and enable PAPs to sense conformational changes in transporters (McNeil et al., 2019; Alav et al., 2021b). In RND efflux pumps, conserved MPD residues such as K366 in AcrA mediate substrate selection. By detecting ligand occupancy in the proximal binding pocket of AcrB, these residues control substrate entry into distinct transport pathways (Alav et al., 2022). Meanwhile, the C-terminal α-helical hairpin of PAPs forms tip-to-tip contacts with the periplasmic opening of outer membrane channels, creating a mechanical interface for signal transmission (Wang et al., 2016a; Ge et al., 2025). Driven by proton motive force (PMF) or ATP hydrolysis, conformational rearrangements in IMPs propagate via the MPD. This induces domain rearrangement in PAPs and functional asymmetry within hexameric subunits (e.g., AcrA/AcrA*), repositioning the C-terminal hairpin. Structural rearrangement further triggers iris-like dilation of the outer membrane channel and initiates substrate efflux (Wang et al., 2016b; Ge et al., 2025). In ABC-type tripartite systems represented by MacAB-TolC, MacA serves as a mechanical transducer that responds to conformational switching of MacB between ATP-bound and nucleotide-free states. This allosteric coupling enhances MacB ATPase activity and transmits periplasmic signals to the cytoplasmic catalytic domain, driving a bellows-like conformational cycle for efflux (Crow et al., 2017).

MFS-family PAPs, exemplified by EmrA, exhibit a unique signal transduction architecture distinct from their RND and ABC counterparts (Hinchliffe et al., 2014; Alav et al., 2021b). The N-terminal transmembrane helix of EmrA carries a leucine zipper motif that drives PAP dimerization and forms a stable binary complex with the transmembrane segments TM1 and TM14 of the MFS transporter EmrB. This arrangement allows EmrB conformational changes to be transmitted into the periplasm (Hinchliffe et al., 2014; Alav et al., 2021b). The conserved RLS motif at the tip of the unusually long α-helical hairpin of EmrA interacts with the VGL motif of the outer membrane channel TolC via tip-to-tip contact. This linkage relays substrate-induced conformational signals from EmrB to open the periplasmic gate of TolC, thereby establishing a continuous trans-envelope efflux pathway (Hinchliffe et al., 2014; Yousefian et al., 2021). In structural assembly, MFS-PAPs assemble into a periplasmic hexameric ring. Head-to-tail arrangement of β-barrel domains forms a sealing structure, while flexible loops of the lipoyl domain constitute a gating ring; this architecture prevents substrate backflow and maintains unidirectional signal propagation (Hinchliffe et al., 2014; Alav et al., 2021b). Unlike RND PAPs such as AcrA, which undergo dynamic conformational cycling and display PAP1/PAP2 structural heterogeneity, MFS-PAPs possess a more rigid α-helical hairpin and a stable hexameric scaffold (Hinchliffe et al., 2014; Alav et al., 2021b). This structural rigidity matches the physiological property that MFS efflux pumps mainly operate as constitutive low-level exporters, rather than stress-inducible high-efflux systems, supporting steady and persistent signal transduction (Alav et al., 2021b).

### 5.3 Substrate specificity, transport, and physiological roles

Building on the structural and signaling roles of PAPs discussed above, we now examine their specific functional contributions to substrate recognition, transport, and broader physiological processes. During substrate recognition and transport, PAPs primarily perform several key roles: (1) They mediate the efflux of harmful substances, thereby preventing their intracellular accumulation and supporting cellular homeostasis (Piddock, 2006; Novelli and Bolla, 2024). (2) They facilitate the removal of metabolites, ensuring that their concentrations remain within appropriate levels (Jang, 2023). Currently identified metabolites transported via PAP-dependent mechanisms include indoles, siderophores, porphyrins, quorum-sensing molecules, pigments, electron carriers, and oligosaccharides (Zgurskaya et al., 2009). Many of these compounds are vital for bacterial survival in specific environments, especially during host invasion and replication. (3) They facilitate the secretion of virulence factors essential for bacterial pathogenicity (Subhadra et al., 2018; Zgurskaya et al., 2009). (4) Furthermore, PAPs participate in “substrate discrimination,” sensing the substrate occupancy status of the IMP to assess substrate suitability and thereby modulate the efficiency and specificity of the efflux process (Alav et al., 2022; McNeil et al., 2019; Symmons et al., 2015).

### 5.4 Interchangeability of PAPs

In *S. enterica*, PAPs such as AcrA, AcrE, MdtA, and MdsA, are essential for bacterial drug resistance and virulence. AcrA and AcrE exhibit a close structural relationship, sharing 69.3% amino acid sequence identity in *S. enterica*, and their predicted secondary structures align closely with the experimentally determined structure of *E. coli* AcrA. Detailed high-resolution structural analysis reveals that each PAP protomer features four interaction sites, corresponding to nine distinct linear “binding boxes,” that are crucial for binding cognate RND transporters (McNeil et al., 2019). The “binding boxes” of AcrA and AcrE are almost identical, and their RND-binding sites are highly conserved, forming the fundamental basis for their functional interchangeability. In contrast, MdtA and MdsA have less than 30% sequence identity with AcrA and AcrE, and their predicted three-dimensional structures are expected to vary significantly. Additionally, the residues within their “binding boxes” that interact with RND transporters differ markedly from those in AcrA and AcrE, influencing their binding specificity. These interaction sites are conserved in spatial position and extent, with specific residues dictating binding preferences and ensuring tight, specific contact interfaces. A predictive model of PAP-RND interactions based on these characteristics reveals the critical factors that influence the occurrence of cross-interactions (McNeil et al., 2019).

Consistent with this structural basis, AcrA and AcrE demonstrate functional versatility across different bacterial species, enabling them to functionally cooperate with various RND transporters. For instance, *S. Typhimurium* AcrA can functionally replace its *E. coli* homolog and effectively partner with the non-cognate RND transporter AcrF (Smith and Blair, 2014). Additionally, AcrE not only partners with AcrB but also establishes a functional relationship with AcrD (Alav et al., 2021a). Since AcrD does not have a cognate PAP, it depends on AcrA to create a functional tripartite efflux system (Yamasaki et al., 2011). In contrast, MdtA and MdsA are limited to interactions with their specific homologous RND transporters (MdtB and MdsB, respectively) and do not engage with non-homologous RND transporters. These findings suggest that the interactions between PAPs and RND transporters are both highly specific yet conditionally promiscuous. The functional redundancy and interchangeability of AcrA and AcrE arise from the high conservation of their RND-binding sites, while MdtA and MdsA’s interactions are restricted to homologous transporters due to structural variations in their binding regions. The potential for PAPs to interchange within efflux systems may endow bacteria with adaptive flexibility needed to resist antimicrobial agents. Furthermore, PAPs are considered promising candidates for the development of inhibitors (Smith and Blair, 2014; Abdali et al., 2017). Future research should delve deeper into the molecular mechanisms governing these interactions, particularly how their dynamic interactions change in response to environmental pressures such as antibiotic exposure and host immune responses, and thereby influence bacterial resistance and virulence.

### 5.5 Signaling from dead cells: the public goods hypothesis

Beyond their canonical intracellular roles, recent evidence suggests that PAPs can also function in the extracellular milieu after cell lysis. When exposed to antibiotics, living bacteria tend to move away from the antibiotic source, indicating that nearby dead cells may emit “necrosignal” that bolster resistance. Research has shown that these deceased cells release a resistance-enhancing factor known as AcrA, which, upon release into the extracellular space interacts with TolC, promoting not only direct drug expulsion but also the upregulation of various efflux pumps and pathways that help combat antibiotic stress, thereby increasing the survival rates of the bacterial community. This process is referred to as “necrotic signal transduction.” As a key periplasmic component of RND drug efflux pumps, AcrA also acts as a necrotic signal that targets homologous pumps. Future studies aim to uncover how AcrA initiates these responses and whether it acts as a signal that prompts the bacterial population to develop an escape strategy against antibiotics (Bhattacharyya et al., 2020; Hofer, 2020).

### 5.6 Functional modulation by environmental signals

Environmental signals regulate PAP function through distinct family-specific mechanisms. In the canonical RND family, AcrA illustrates this principle. The conformational changes induced by pH and Mg^2 +^ (detailed in Section 3) directly tune the function of the AcrAB-TolC pump. This allosteric control adjusts efflux efficiency and substrate range across physiological pH values, ensuring survival in acidic niches or fluctuating metal conditions (Lewis et al., 2025). Similarly, CusB undergoes a specific conformational rearrangement upon high-affinity binding of Ag^+^/Cu^+^. This metal-induced activation directly switches on the efflux channel, defining a substrate-driven functional output (Su et al., 2009; Bagai et al., 2007).

In contrast, other PAP families rely more heavily on indirect, transcriptional adaptation. For example, MacA functions independently of pH changes at the protein level. Instead, under low Mg^2 +^ conditions, the PhoPQ two-component system upregulates the *macAB* operon, boosting macrolide efflux during nutrient scarcity (Nagano et al., 2019). Likewise, MdtA lacks obvious pH-sensing domains. However, alkaline pH strongly induces *mdtABC* expression via the CpxAR system, converting envelope stress into increased pump production (Gerken and Misra, 2010). For MFS-family PAPs (e.g., EmrA and EmrK), direct cation sensing remains uncharacterized. Their sensitivity to pH likely stems from dependence on the proton motive force (PMF) or regulation by global transcriptional factors like MarA and Rob.

In summary, PAP regulation is highly diverse. From the direct conformational tuning of AcrA and CusB to the transcriptional remodeling seen in MacA, MdtA, and EmrA, each family has evolved unique strategies tailored to its energy source and primary substrates. It would be inaccurate to view pH and cation sensing as a universal mechanism governing all PAPs.

## 6 Epidemiological evidence of PAPs in clinical resistance

Although missense or nonsense mutations in PAP-coding genes are rarely reported as direct drivers of resistance, epidemiological data from clinical isolates indicate that PAP overexpression is the predominant mechanism underlying high-level drug resistance.

*A. baumannii* exemplifies this trend. While structural mutations in *adeA* (encoding the AdeABC PAP) are sporadic, overexpression of AdeABC is prevalent in multidrug-resistant (MDR) and extensively drug-resistant (XDR) isolates. This upregulation is typically driven by mutations in the AdeRS two-component system and correlates with elevated MICs to last-resort antibiotics like tigecycline (Sun et al., 2014; Yoon et al., 2013; Rumbo et al., 2013).

Similarly, in *K. pneumoniae*, resistance stems more from efflux system dysregulation than from structural PAP mutations. Clinical strains frequently overexpress *acrA* (AcrAB-TolC) or *oqxA* (OqxAB), resulting in decreased susceptibility. These changes are often attributed to mutations in upstream regulators such as *ramR* or *acrR* (AlMatar et al., 2025; Bialek-Davenet et al., 2015).

In *E. coli*, hyperproduction of AcrA has been documented in clinical isolates with high-level fluoroquinolone resistance (Li et al., 2005). More recently, *acrA* upregulation was identified in carbapenem-resistant *E. coli* subpopulations displaying heterogeneous resistance to tigecycline (Liu et al., 2021).

Collectively, these findings demonstrate that PAP-mediated resistance relies primarily on transcriptional overexpression governed by regulatory mutations or stress pathways, rather than on structural alterations of the PAP proteins themselves. This distinction underscores the critical role of transcriptional control in the clinical evolution of efflux-associated antimicrobial resistance.

This does not diminish the importance of PAPs as dynamic regulators; rather, it suggests that dysregulation of their expression, rather than disruption of their allosteric mechanisms, is the primary route to clinical resistance. Whether small-molecule targeting of PAP allostery can overcome this expression-driven resistance remains an open question.

## 7 PAPs as targets for EPIs

Efflux pump inhibitors represent a promising new therapeutic approach that aims to enhance the effectiveness of current antibiotics and mitigate the rise. The AcrAB-TolC system is a primary focus for these inhibitors (Vieira Da Cruz et al., 2024; Pérez et al., 2012). Earlier studies have mainly concentrated on the IMP AcrB, which several EPIs have been shown to target (Opperman and Nguyen, 2015). Nevertheless, the clinical translation of these EPIs has been hindered by toxicity issues and the broad substrate specificity of AcrB, which transports a wide range of compounds (Abdali et al., 2017). Consequently, attention has shifted toward alternative targets within the efflux apparatus.

Periplasmic adaptor proteins play a vital role in efflux pumps by facilitating the linkage between outer membrane channels and transmembrane complexes, and are increasingly recognized as promising inhibitory targets. Abdali et al. identified eight EPIs, including NSC60339, that bind to AcrA. These compounds alter the structure of AcrA *in vivo* and enhance the antibiotic efficacy against E. coli and other Gram-negative pathogens (Abdali et al., 2017). Studies have shown that NSC60339 acts as a molecular wedge within the gap between AcrA’s lipoyl and β-barrel domains, which disrupts the drug-induced signal transmission from AcrB to TolC (Lewis et al., 2023). This disruption could hinder AcrA’s ability to respond to changes in the periplasm and to relay conformational shifts from AcrB to TolC (Abdali et al., 2017).

Notably, AcrA belongs to a broader family of RND-associated PAPs. Due to the functional overlap between AcrA and its homolog AcrE, developing inhibitors that can effectively target both the proteins simultaneously is crucial. This approach would improve antibiotic sensitivity, reduce virulence, and help prevent the emergence of resistance (McNeil et al., 2019). Furthermore, designing targeted inhibitors against PAPs or ternary complex assembly, based on the key residues identified in RND binding sites, could yield innovative strategies to tackle bacterial infections and develop novel antimicrobial therapies.

At present, the clinical application of AcrB-targeting EPIs is limited by inherent challenges, while EPIs aimed at PAPs, such as those targeting AcrA, show potential, with their mechanisms already partially understood. Future investigations should focus on further characterizing these targets and optimizing their inhibitors, paving the way for novel strategies to combat antimicrobial resistance.

## 8 Conclusions and future perspectives

### 8.1 Concluding remarks

This review outlines the updated understanding of PAP function. These proteins are no longer regarded as static structural scaffolds, but as dynamic hubs for allosteric regulation (Zgurskaya et al., 2015). We summarize three key findings from current evidence. First, the inherent structural features of PAPs support conserved periplasmic bridging interactions, while allowing functional flexibility across bacterial systems. Second, PAP regulatory patterns vary considerably among efflux pump families. In RND efflux systems, PAP function is modulated through direct physicochemical interactions (Su et al., 2009; Lewis et al., 2025). In ABC and MFS systems, PAP activity is mainly controlled via transcriptional reprogramming. Third, clinical antibiotic resistance driven by PAPs arises predominantly from elevated PAP expression caused by regulatory changes, rather than structural gene mutations (Sun et al., 2014; Yoon et al., 2013; AlMatar et al., 2025). Collectively, these findings establish PAPs as critical functional hubs that support bacterial adaptive responses.

### 8.2 Future perspectives

Future work can focus on two key research directions: basic mechanistic study and translational application research. Further mechanistic studies need to clarify how PAPs sense and respond to host microenvironmental cues including immune factors and nutrient scarcity. It is also essential to define the molecular basis for conserved and species-specific PAP-RND binding, and to explore how such protein interactions evolve under long-term selective pressure.

Translational research favors PAPs over IMPs as drug targets. More efforts should focus on developing broad-spectrum PAP inhibitors rationally. Researchers can target cryptic pockets in PAPs to block allosteric signaling with their cognate transporters. Future EPI development should move beyond single-PAP targeting (e.g., AcrA alone) toward dual inhibitors of AcrA and AcrE, using their conserved binding motifs. Additionally, targeting PAP–OMF interfaces or disrupting dynamic assembly represents an underexplored avenue. Such strategies may reduce drug toxicity and limit bacterial resistance.
